# Two mini transverse-incision repair yields better results than percutaneous repair for acute closed midsubstance Achilles tendon rupture: a retrospective case-control study

**DOI:** 10.1186/s13018-024-04904-8

**Published:** 2024-07-31

**Authors:** Wen Tao Jin, Li Fang Huang, Hai Hua Guo, Lei Wang, Xiang Li, Ze Jin Wang

**Affiliations:** Sports Medicine Division, Department of Orthopaedics and Traumatology, The University of Hong Kong-Shenzhen Hospital, Shenzhen, China

**Keywords:** Achilles tendon rupture, Minimally invasive repair, Mini open repair, Percutaneous repair

## Abstract

**Background:**

Acute closed midsubstance Achilles tendon rupture(ACMATR) is common, with various treatment methods developed over time. We retrospectively compared the two mini transverse-incision repair (2MTIR) with percutaneous repair (PR) to determine which method yields better results.

**Methods:**

All cases meeting criteria from 2018 to 2021 in our hospital were included and followed up for 1 to 5 years. A final questionnaire with multiple indexes was conducted via phone call. Comparative analysis of these indexes between the two groups was performed using IBM SPSS Statistics (V.26). Continuous variables that passed tests for normality and equal variance were compared using the Student’s t-test. Ranked data were compared using the Mann-Whitney U test. Categorical variables were tested with the chi-square test or Fisher’s exact test. A p-value of less than 0.05 was considered statistically significant.

**Results:**

There was one rerupture in the PR group. The final indexes for “Tightness Feeling”, “Heel Rising Strength”, and “Foot Numbness” were statistically different (*P* < 0.05) between the two groups. The “Re-rupture” and “Return to Sports” indexes showed no statistical difference (*P* > 0.05).

**Conclusions:**

The 2MTIR technique provided a technically straightforward, minimally invasive procedure with well-preserved paratenon and direct end-to-end firm fixation in cases of ACMATR. It resulted in very low complications, easy rehabilitation, and full weight-bearing as early as 5–6 weeks postoperatively, yielding better functional outcomes compared to the PR technique in the 1–5 year follow-up.

**Trial registration:**

The study was preliminarily registered and approved by the University of Hong Kong-Shenzhen Hospital Ethical Board with Project number: hkuszh2023074 on May 4, 2023.

## Background

Acute closed midsubstance Achilles tendon rupture (ACMATR) is common in clinical practice. Studies have identified various factors that can predispose the Achilles tendon (AT) to injuries and rupture. Chronic repeated microinjuries and corticosteroids, administered either systemically or locally in the region of the AT, have been proven to be associated with AT rupture [1]. Additionally, there is a documented association between AT rupture and sciatica [2]. Fluoroquinolones have also been shown to have a close relationship with AT disorders [3]. Typically, acute ruptures of the AT occur in men in their third or fourth decade of life, who work in white-collar professions and occasionally play sports [1]. The longstanding debate over the relative merits of surgical repair versus conservative treatment has been ongoing [1, 4–9]. Various techniques for surgical repair have been published in the literature. The goal of treatment is to enable patients to return to their pre-injury level of activity, especially sports, with minimal sequelae. Both conservative treatment and surgical repair can present complications. For conservative treatment, the main concerns are the re-rupture rate and tendon lengthening after healing, which can result in calf weakness. This may be due to gapping or poor opposition of the rupture ends, and prolonged immobilization leading to irreversible muscle atrophy [1, 4–7]. Surgical intervention, particularly the traditional medial longitudinal incision open repair, has a higher rate of postoperative wound problems and can result in tightness or stiffness [1]. Minimally invasive techniques such as percutaneous [10–14] and mini-open repairs [15–18], typically result in a very low rate of wound problems and less interference with the tendon’s blood circulation. However, percutaneous repair may have a higher rate of sural nerve injury, and some techniques require special jigs [15, 19, 20] to capture the rupture ends and guide the sutures, which may not provide as strong a purchase on the tendon substance as open techniques. These specialized surgical kits are not available in every hospital. From the surgeon’s perspective, stronger repairs provide more confidence for earlier and more aggressive rehabilitation, which benefits those actively engaged in sports. Evidence shows that reinjuries of the AT are more common in some athletes after an early return to play [21].

The drawbacks of each method lead to treatment diversification. Ideally, a simple technique would approximate the rupture ends firmly without easy gapping, require minimal rehabilitation, avoid wound infections and sural nerve injury, and achieve good heel rising strength and normal range of motion (ROM) compared to the other side as early as possible.

From 2018 to 2020, we primarily treated indicated ACMATR with percutaneous repair (PR). From late 2019 onwards, we gradually shifted to two mini transverse-incision repair (2MTIR). In this study, we retrospectively compare the results between these two groups after 1–5 years of follow-up to determine which method yields better outcomes.

This study was approved by the hospital’s ethical administration.

## Materials and methods

This is a retrospective case-control study. The re-rupture rate and some subjective indices were compared statistically between the PR and 2MTIR groups.

### Patient selection

All ACMATR cases admitted to our hospital and treated surgically from 2018 to 2021 were investigated. The inclusion criteria were: 1)Primary acute injury involving the AT during sports or a fall. 2) Symptoms included weakness and pain in the ankle. 3) Physical examination showed no wound and good skin condition, but an obvious empty gap was palpable in the AT region with no pain on the calcaneus tubercle, and a positive Thompson sign. 4) X-ray showed no avulsion fracture of the calcaneal tuberosity. 5) Injury time was less than 7 days. 6) Surgery confirmed a complete rupture in the midsubstance of the AT without involvement of tendon avulsion from the calcaneal tuberosity. The exclusion criteria were: 1)History of chronic systemic disease requiring systemic administration of corticosteroids or fluoroquinolones from 6 months prior to injury until last follow-up. 2)History of corticosteroid injection in the AT region. 3) Presence of sciatica symptoms at the time of injury. 4) Previous rupture of the opposite side AT. 5)Other diseases that prevented patients from performing rehabilitation exercises.

### Surgical technique

#### General preparation

All surgeries were performed by the same team of five surgeons at the same hospital. After spinal or general anesthesia, the patient was placed in a prone position with the knee extended, and the resting ankle angle was estimated on the healthy side. The surgical side was scrubbed and draped as usual. The sural nerve was marked according to general anatomy: at 9.8 cm proximal to the calcaneus, the nerve crosses the lateral border of the AT. Distally, the nerve passes laterally, so that it is 18.8 mm lateral to the lateral border of the AT at the level of AT insertion into the calcaneus [14].

#### Tendon repair sutures

Two different color stitches were used, selected from two packages of #2 ORTHOCORD (one violet and one blue braided composite suture, Le Locle, Switzerland), preparing a total of four strands of sutures.

#### Group 1 - PR group

**Fig. 1:**
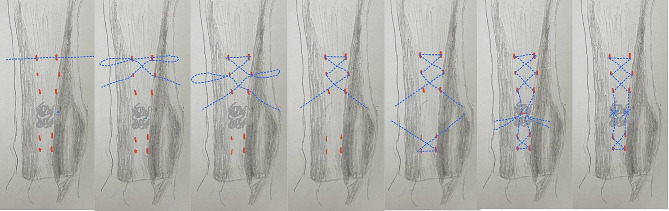
Surgical procedure step by step. *Keys* Red Dash ---- Stab Wound, Blue Dots Line --- Suture, Blue Cross ---- Knots

The rupture gap and two stumps were palpated. To weave the tendon as shown in Fig. 1, a 2 mm stab wound was first created at the planned puncture site. A small, curved mosquito was used to open the subcutaneous tissue, and then a #16 gauge needle was used to puncture the tendon in the center, confirming the puncture by feeling resistance. A #0 ETHICON PDS II suture was guided through the needle, and then an ORTHOCORD suture was threaded through the tendon using the PDS. This process was repeated according to the route shown in Fig. 1, creating a suture pattern similar to a Bunnell stitch. At the center of the gap, a 1–2 cm transverse incision was made with a #11 blade, and the paratenon was incised transversely by 1 cm. Typically, there were six stab wounds on the proximal stump side and four on the distal stump side. In each stump, two sutures were passed following the same steps, with four ends emerging from the central open incision. The two stumps were then pulled tightly towards the gap to test the purchase of the sutures in the tendon. If the purchase was satisfactory, two pairs of sutures were preliminarily tightened, and the tension of the knots was adjusted according to the resting ankle angle compared with the healthy side, usually set at 20–30 degrees of plantar flexion. Finally, all four pairs of sutures were securely tightened. The stab wounds were usually closed with adhesive strips, and the central incision was closed with sutures for the paratenon, subcutaneous tissue, and skin. A short ankle cast was applied to fix the ankle at 20–30 degrees of plantar flexion. The cast was removed two weeks postoperatively, and the wound was inspected. If no issues were found, another cast was applied and left in place until six weeks postoperatively without further change.

#### Group 2–2MTIR group

**Fig. 2:**
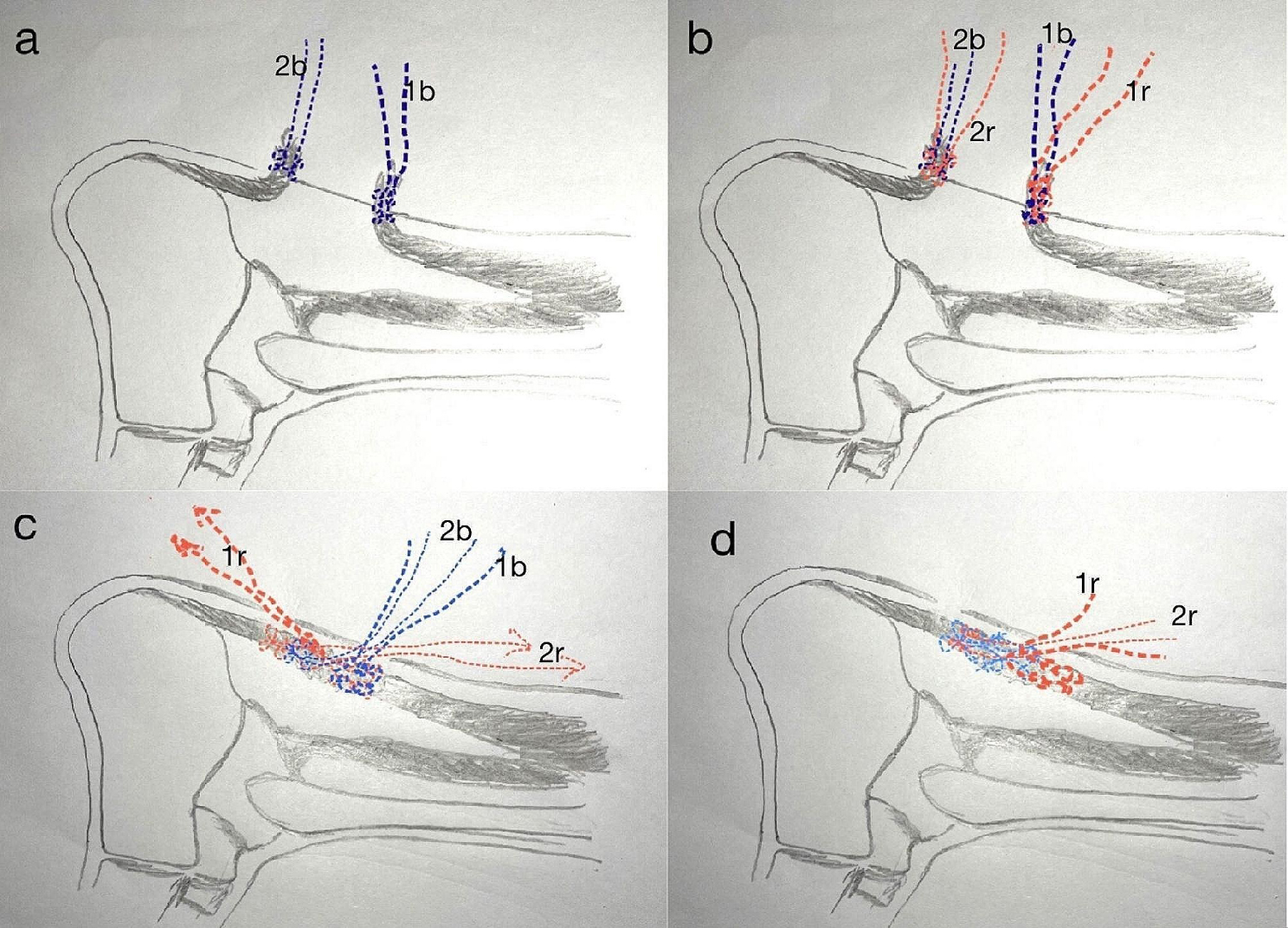
The 2MTIR technique. (**a**) Two stumps were exposed through separate transverse mini incisions. One suture (1b and 2b) was placed in a Krackow pattern in each of the stumps. (**b**) A second, different color suture (1r and 2r) was placed in each of the stumps. (**c**) Sutures 1r and 2r were threaded through the tunnel to the opposite incision to pull the two stumps together, and the 2b suture ends were threaded through the tunnel to the proximal incision and tied in knots with 1b. (**d**) The 1r ends were threaded to the proximal incision and tied in knots with 2r

**Fig. 3:**
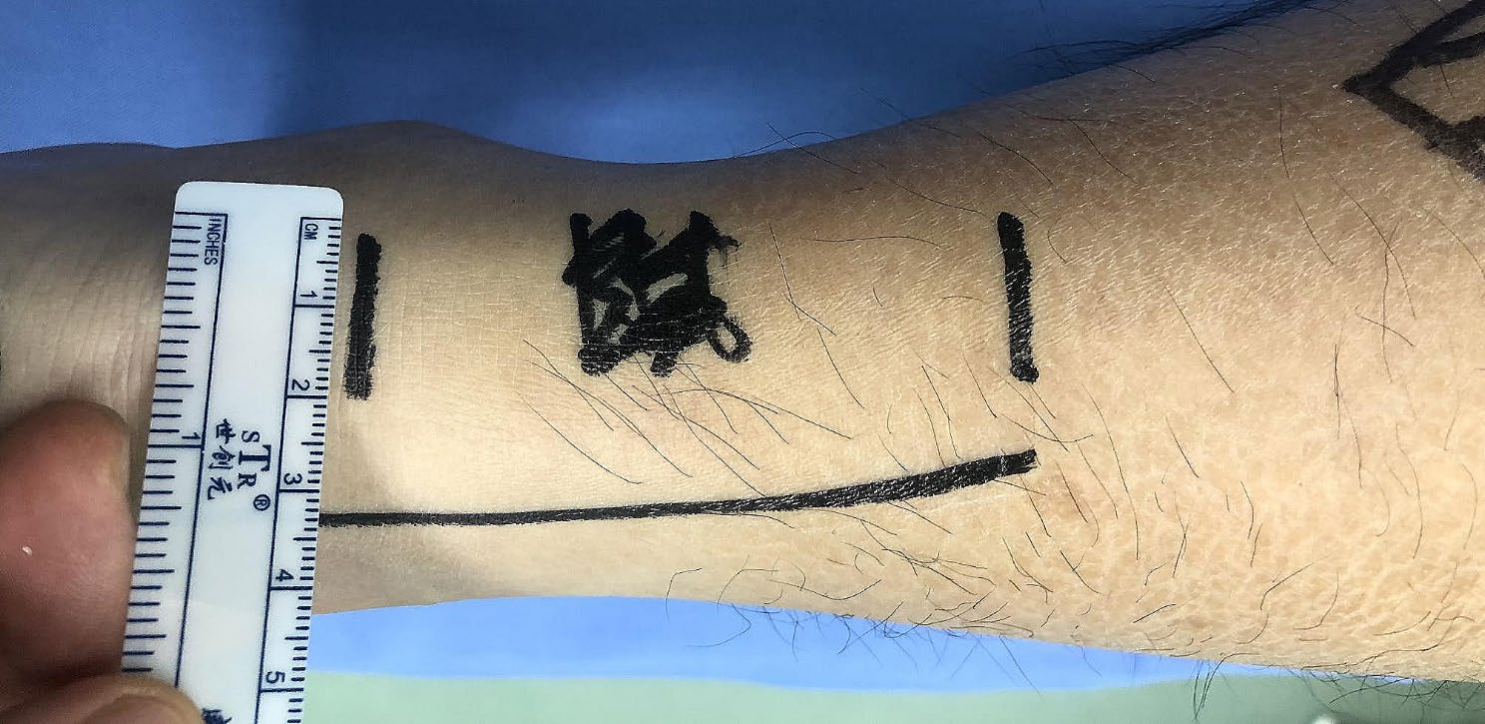
Incision

**Fig. 4:**
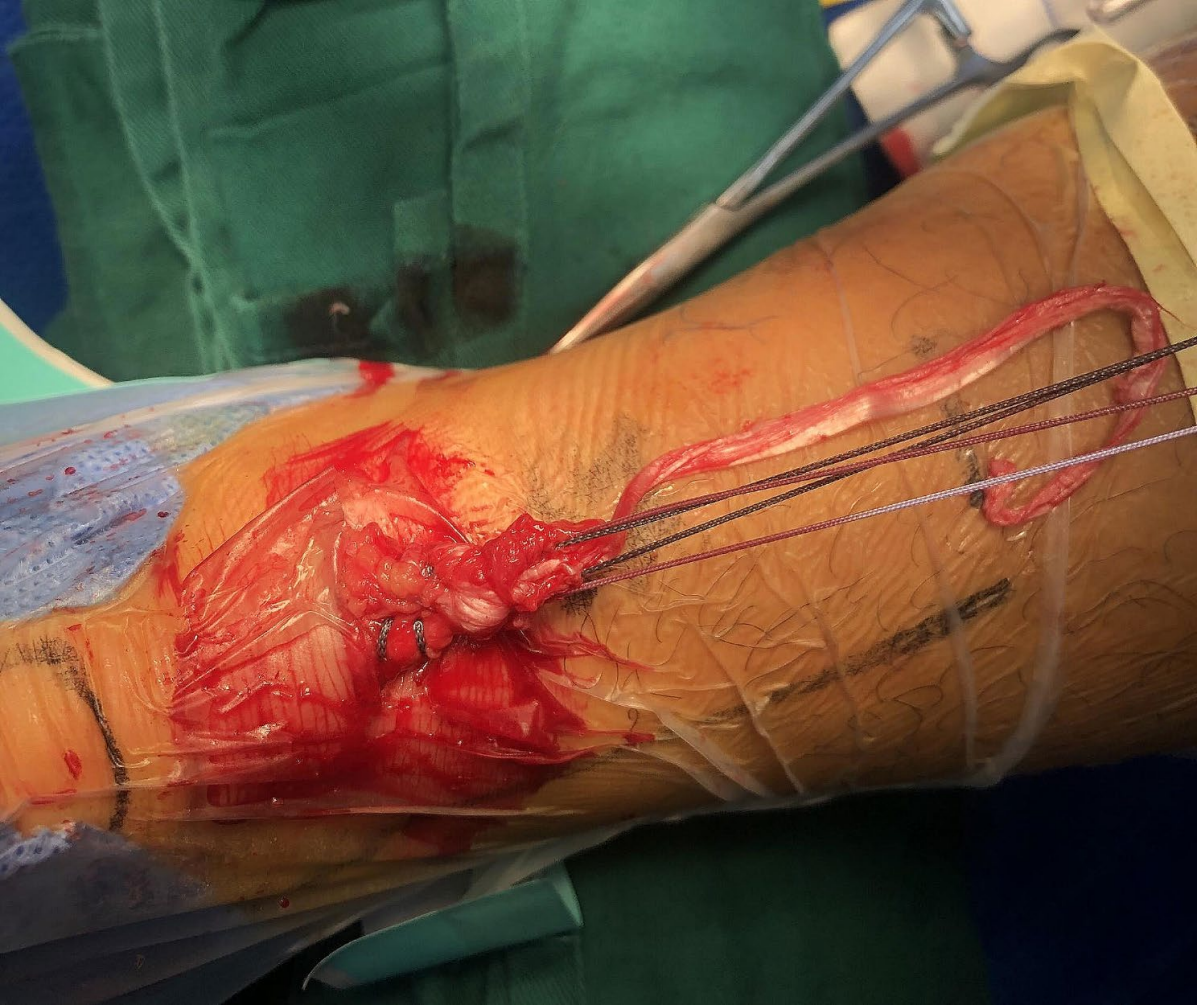
The distal rupture end was exposed, and two Krackow-type sutures were placed in the stump

**Fig. 5:**
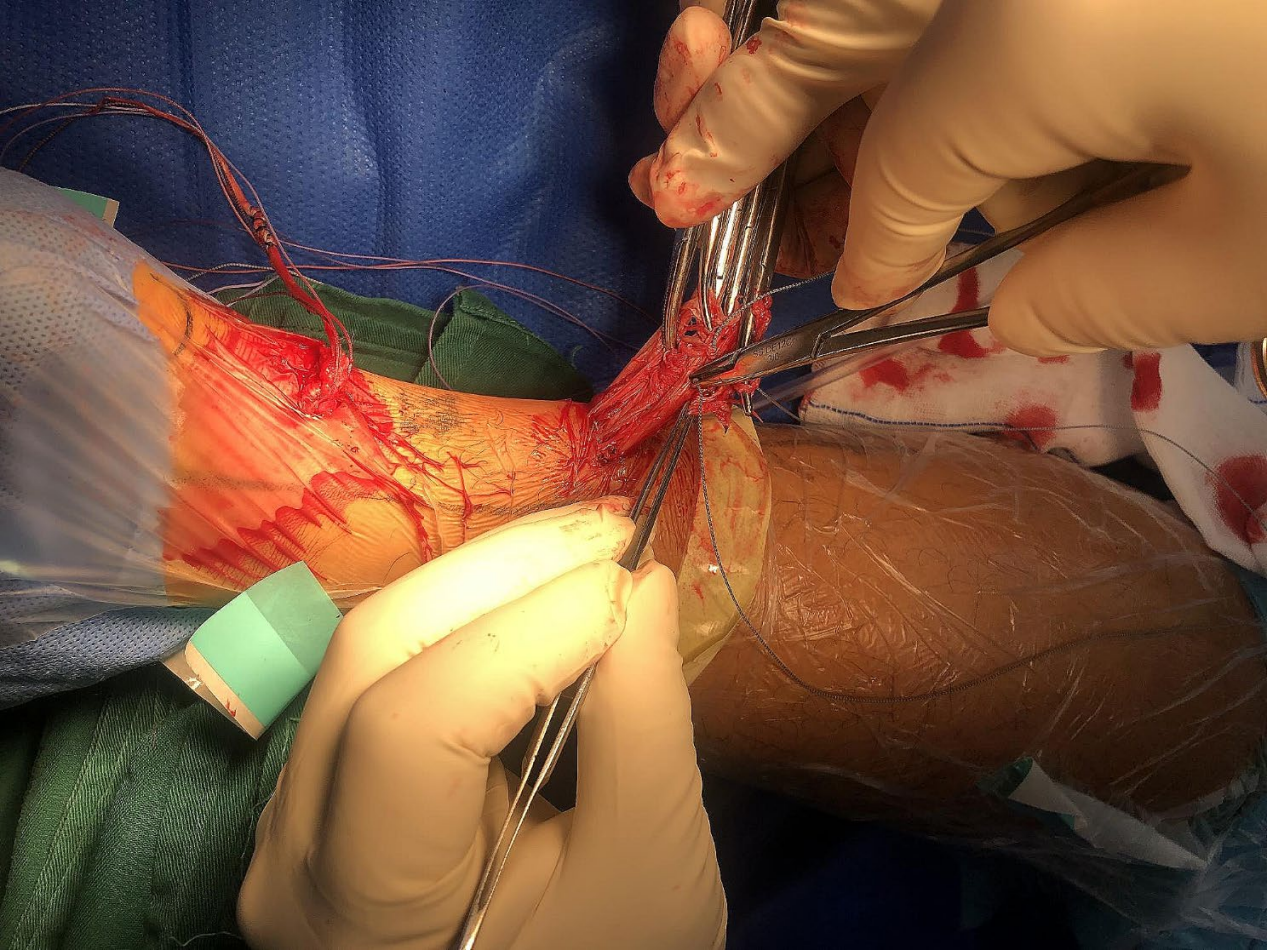
The proximal rupture end was exposed, and sutures were placed

**Fig. 6:**
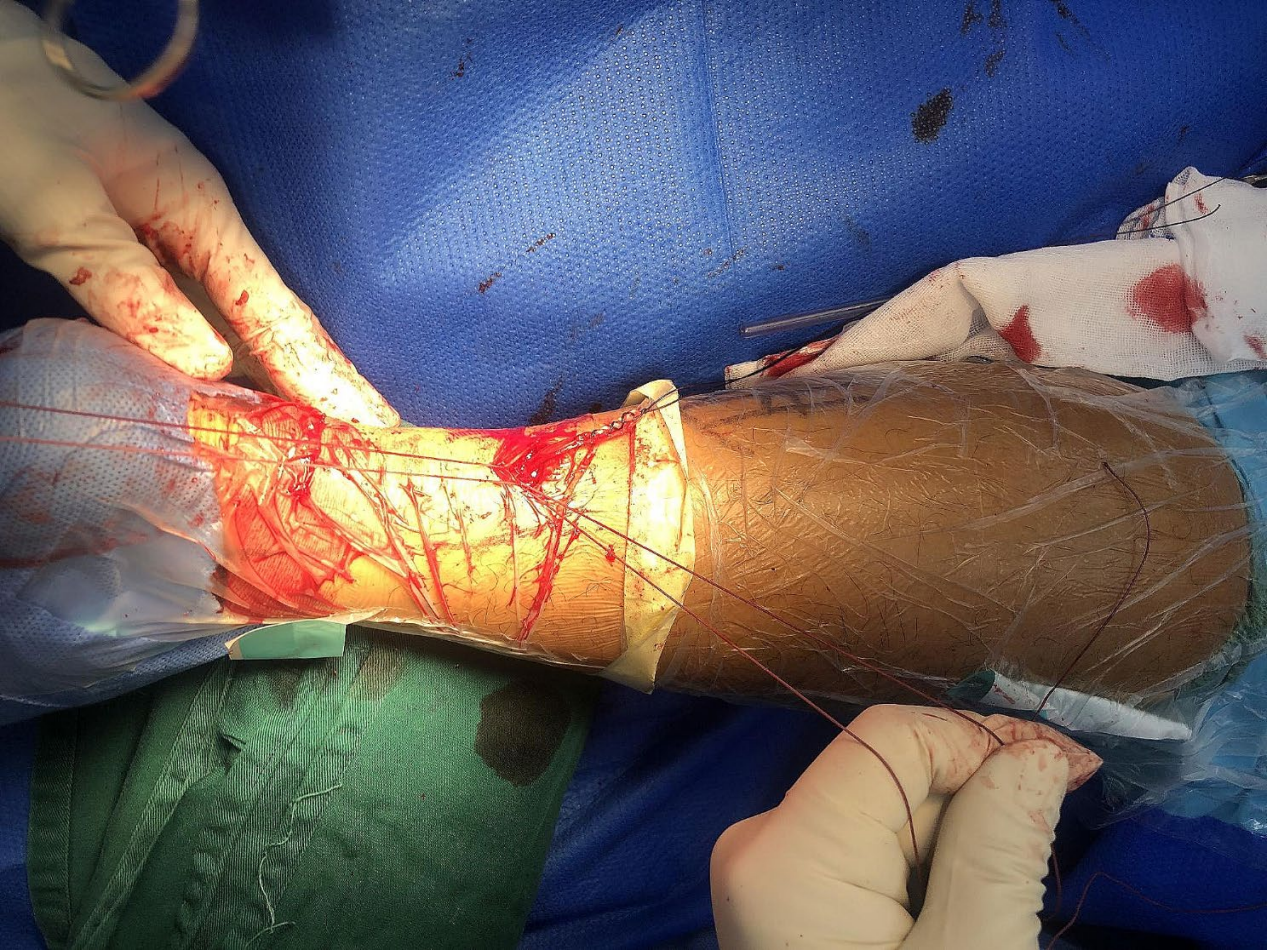
One pair of sutures was tightened

The steps are illustrated in Figs. 2, 3, 4, 5 and 6. The gap between the two rupture ends was palpated, typically ranging from 3 to 5 cm. Two transverse incisions, each 2 cm in length, were made. The distal incision was usually 2 cm distal to the palpated distal rupture stump, right in the center of the tendon. The paratenon was incised, and artery forceps were used to hook out the distal remnant. With the ankle in a plantar flexion position, two different color braided sutures were placed into the distal rupture end using a technique similar to the Krackow stitch, resulting in four suture ends. The second transverse incision, also 2 cm in length, was made about 2 cm proximal to the margin of the proximal rupture stump, at a location with good tendon substance. This proximal incision was typically about 4–8 cm apart from the distal one and needed to be relatively medial to avoid the sural nerve. The skin was incised, and the subcutaneous tissue was divided from medial to lateral, carefully looking for the sural nerve, which is longitudinally arranged and may crosses on the lateral side of the incision. We seldom encountered the sural nerve in this area. The paratenon was incised transversely, and multiple artery forceps and knee flexion were used to carefully retrieve every scattered proximal tendon fiber, some of which were often contracted far proximally. Up to five artery forceps were necessary to fish out all the fibers hiding in the proximal paratenon. The rupture end was then neatly combed. Two similar Krackow stitches were performed on the proximal end using two different color sutures, as done for the distal end. Since rupture fibers are always at different levels, the braided sutures on both the proximal and distal rupture ends should not reach the very end of the longest fibers. If they did, the tendon would be lengthened after the knots were tightened. The stitches should only go through the relatively robust part of the tendon that could provide a good hold for the sutures. With the two rupture ends exposed, a suture retriever was used through the paratenon tunnel between the two incisions to bring one pair of distal sutures out of the proximal incision and one pair of proximal sutures out of the distal incision. These two pairs of sutures were used to pull the two rupture stumps together. The other pair of sutures from the distal end was then retrieved out of the proximal incision. With the first two pairs of sutures pulling to the opposite sides, aiming for an ankle plantar flexion of 20–30 degrees, the other two pairs of sutures were tightened with a knot pusher through the proximal incision. Then, for the first two pairs of sutures, one pair was retrieved to the same incision as the other pair and tightened in the same way. The stitches were carefully managed to avoid twisting. The knots were usually located within the paratenon and in the central tunnel between the two incisions. The paratenon was repaired in both incisions with absorbable sutures (3/0 Vycryl), typically one or two stitches per incision. The skin was then closed. A short cast was applied at the same angle of ankle plantar flexion after the repair. The cast was opened at 2 weeks postoperatively, and the stitches were removed. The repaired tendon was palpated, usually feeling thick, sturdy, and continuous without any thinner parts. A new cast was applied, and by 4 weeks postoperatively, the cast was removed.

### Patient follow-up

Patients in both groups were usually discharged from the hospital on the second day after surgery and rechecked at 2-week intervals until 2–3 months postoperatively, followed by monthly visits for another 2–3 months. They did not attend a rehabilitation center; instead, rehabilitation was mostly self-administered. Surgeons instructed patients on how to use crutches for walking and to perform ipsilateral hip, knee, and toe exercises frequently on an hourly basis while awake. Patients were advised to elevate their legs to heart level to prevent swelling. After cast removal in both groups, patients were taught to perform active ankle flexion and extension exercises for 5 min every 1–2 h as tolerated by pain. Tiptoe weight-bearing with a heel-raised boot and crutches was initiated. The height of the heel wedges was adjusted so that some tightness of the repaired AT could be felt while walking. The heel wedges were gradually decreased, one every 2–4 days. When the ankle could be placed in a neutral position without excessive tightness of the AT, the heel-raised boot was discontinued, and sports shoes were encouraged for full weight-bearing walking, which usually occurred at 8–10 weeks postoperatively in the PR group and 5–6 weeks in the 2MTIR group. By 3 months postoperatively, patients were encouraged to begin jogging if it was tolerated. By 4–6 months, they could resume sports depending on their personal perception of the repaired tendon’s strength and pain levels.

**Fig. 7:**
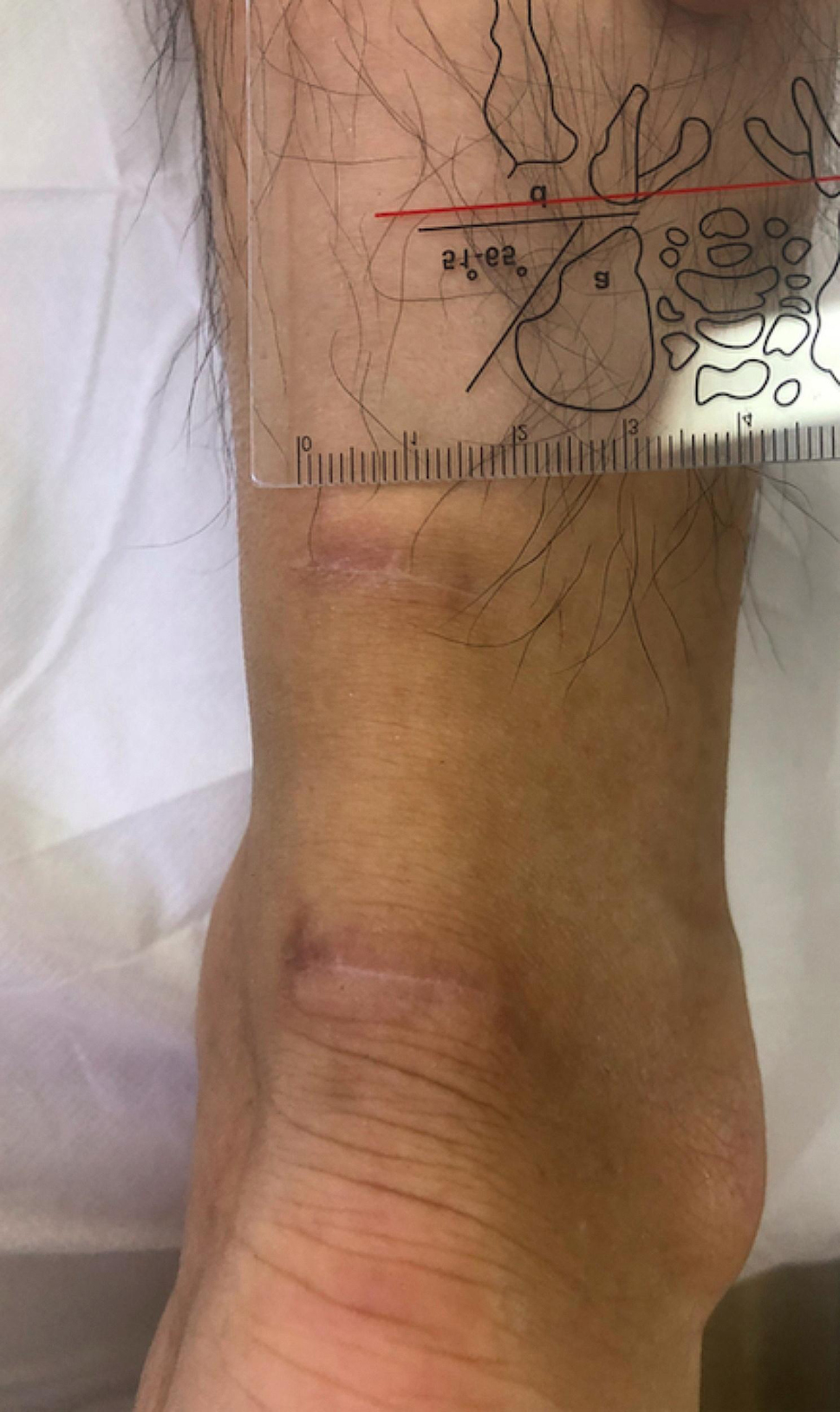
One of the typical 2MTIR group patients 6 months postoperatively

The biological, surgical, and early stage clinical follow-up data (usually until 6 months postoperatively) were collected from the hospital HIS system. The final follow-up (1–5 years postoperatively) was mostly conducted via phone. A typical final healing picture in the 2MTIR group is shown in Fig. 7.

### Final follow-up questionnaires

The final follow-up questionnaires are listed in Table 1. These questions are subjective. The sensation of tendon tightness during a full deep squat can indicate if there is an ankle dorsiflexion deficit due to shortening the AT. Conversely, the absence of tightness might suggest possible tendon lengthening. This feeling should be compared with the normal side. Heel rising strength tests active muscle power recovery. Return to sports(RTS) represents the general recovery of patients. Re-rupture and foot numbness are both considered complications. Phone calls were made to the cases, and each question was asked. Answers were documented for statistical analysis.

**Table 1:** Questionnaires for final follow-up

1) Tightness Feeling	a. No tightness when fully deep squatting
	b. Yes, but can still fully deep squat
	c. Cannot fully deep squat
2) Heel Rising Strength	a. Up to 80% of the opposite side
	b. Up to 50–80% of the opposite side
	c. Less than 50% of the opposite side
3) RTS	a. Engages in activities like badminton, soccer, basketball, or tennis (requiring a lot of pivoting and jumping with strong plantar flexion power)
	b. Engages in activities like jogging, cycling, or swimming (requiring moderate ankle plantar flexion power)
	c. Engages in activities like walking and hiking (requiring mild ankle plantar flexion power)
4) Re-rupture	(a) Yes (b) No
5) Foot Numbness	(a) Yes (b) No

### Statistics

IBM SPSS Statistics (Version 26) was used for analysis. Continuous variables that passed tests for normality and equal variance were compared between groups using Student’s t-test. Ranked data were compared between groups using the Mann-Whitney U test. Categorical variables were tested with the chi-square test or Fisher’s exact test. A p-value of less than 0.05 was considered statistically significant.

## Results

1. Thirty-one cases in the 2MTIR group and thirty-seven cases in the PR group were collected from the HIS system. The existing hospital follow-up records (usually lasting until 3-6months postoperatively) show no infection or other skin complications. However, five cases in the 2MTIR group and three cases in the PR group could not be contacted for the final follow-up despite more than ten phone calls over ten days. The existing medical records in the HIS for these eight cases were carefully inspected with no evidence of other complications. These eight cases were excluded from the final comparison test.
2. The biological data and re-rupture rates are listed in Table 2, showing no statistical difference between the two groups (*P* > 0.05).
  One re-rupture occurred in the PR group. The patient was 28 years old and experienced a re-rupture two months after the initial surgery due to a slip and fall while walking. He felt pain in the AT. A physical exam and B ultrasound were performed, followed by an open medial longitudinal incision surgery, which confirmed the re-rupture at the same location as the first surgery. After scar resection, end-to-end suturing was not possible, so a graft bridging with the semitendinosus was performed to bridge the gap.

3. The re-rupture case was then excluded from the PR group. For the remaining 26 cases in the 2MTIR group and 33 cases in the PR group, the 1–5 year(20 months − 5 years) follow-up rates were 26/31 (84%) in the 2MTIR group and 33/37 (89%) in the PR group.
4. For the final follow-up questionnaires (Table 1), data were collected and compared between the two groups. Statistical results are listed in Table 3. Significant differences were found in tendon tightness, heel rising strength, and foot numbness (*P* < 0.05), while there was no significant difference in RTS (*P* > 0.05).

**Table 2:** Gender, age, and re-rupture comparison between 2 groups

Variables	2MTIR(n = 26)	PR(*n* = 34)	t value	*P* value
Gender			-	> 0.999
Male	26(100.00%)	33(97.06%)		
Female	0(0.00%)	1(2.94%)		
Age	37.42 ± 6.47	35.71 ± 4.20	1.177	0.246
Re-rupture			-	> 0.999
Yes	0(0.00)%	1(2.94%)		
No	26(100.00%)	33(97.06%)		

**Table 3:** Comparison between the two groups excluding re-rupture case

Variables	2MTIR(n = 26)	PR(*n* = 33)	t/Z/χ2	*P*
Gender			-	> 0.999
Male	26(100.00%)	32(96.97%)		
Female	0(0.00%)	1(3.03%)		
Age	37.42 ± 6.47	35.94 ± 4.04	1.023	0.313
Tightness Feeling			2.477	0.013
a	11(42.31%)	24(72.73%)		
B	13(50.00%)	9(27.27%)		
c	2(7.69%)	0(0.00%)		
Heel Rising Strength			-3.211	0.001
a	25(96.15%)	19(57.58%)		
b	0(0.00%)	11(33.33%)		
c	1(3.85%)	3(9.09%)		
RTS			-	0.923
a	15(57.69%)	20(60.61%)		
b	9(34.62%)	10(30.30%)		
c	2(7.69%)	3(9.09%)		
Foot Numbness			4.393	0.036
yes	0(0.00%)	7(21.21%)		
No	26(100.00%)	26(78.79%)		

## Discussion

Various treatments for ACMATR are widely published in the literature, ranging from conservative treatment to many minimally invasive techniques with or without specially designed surgical jigs [1, 4–12, 15, 19, 20]. The common goal is to minimally disrupt blood supply to the rupture stumps and maintain an intact paratenon while ensuring good tendon stump approximation to promote early healing and rehabilitation. Consequently, earlier and better functional recovery without complications is expected. Across different studies, the time to full weight bearing without protection varied from 6 to 12 weeks postoperatively [10–15, 18, 22, 23]. Conservative treatment and less robust minimally invasive techniques necessitate a longer and slower rehabilitation protocol to prevent lengthening or re-rupture of the AT.

The technique we used in the 2MTIR group is similar to that described by Kord MC et al. [18]. In their practice, they used two longitudinal incisions and the “gift box technique”, avoiding the knots being at the junction of the two rupture ends, whereas we utilized two transverse ones. The advantage of our approach is better preservation of the paratenon and improved cosmetic outcomes. The technique used in the PR group is similar to that originally used by Ma GW et al. [10].

Our study found significant differences in the indices of tightness feeling, heel rising strength between the two groups, indicative of better tendon length preservation in the 2MTIR group. There was one case of re-rupture in the PR group, but no statistical difference.

Firstly, regarding fixation strength and healing patterns, based on our surgical experience, the opposition accuracy of the two rupture ends is much better in the 2MTIR group than in the PR group. It is rare for the rupture ends to align completely without a gap simply after ankle plantar flexion in sports injury cases. We observed that in most 2MTIR cases, the rupture fibers in the proximal ends contracted unequally, with some fibers retracting far from the rupture margin. During surgical repair, these further retracted fibers were carefully pulled out of the proximal stump and properly aligned before suturing. In the PR group, due to limited exposure, only some of the fibers that did not retract far away could be pulled into contact, while the remaining fibers that retracted more proximally could not reach their counterparts. The suture method also differed between the groups: the Krackow technique in 2MTIR under direct vision and the Bunnell technique in PR without direct vision. This difference in technique resulted in varying tendon tissue purchase strengths. Watson et al. [24] demonstrated that the Krackow locking repair is stronger than the Bunnell technique. Considering the contact area between the tendon ends and the suture technique, the repaired tendon is stronger in the 2MTIR group than in the PR group. Bae et al. [25] reported that among 15 cases of postoperative AT infection treated with debridement, resection of unhealthy tissue, and cast fixation accompanied by functional rehabilitation, 10 cases were capable of performing a single-limb heel rise at the final follow-up (22–97 months). This extreme example demonstrates that fibrous scar healing with a large tendon defect is possible in ruptured AT. The high success rate of conservative treatment [4, 5, 8, 9, 23] also suggests that as long as the paratenon remains intact with a good blood supply, the AT has a high healing potential, even if there is a gap between the rupture ends. Under protection, fibrous scar within the paratenon can eventually remodel and adhere to both stumps, restoring continuity even if the two stumps were not originally in good contact. Tendon healing is characterized by the formation of fibrovascular scar tissue [26], which involves a combination of extrinsic and intrinsic mechanisms [27]. Currently, extrinsic-predominant healing, associated with larger tendon diameter and more adhesions, is considered the main form of healing in AT ruptures [26, 28]. A well-preserved paratenon is crucial for extrinsic healing to occur [26]. Intrinsic healing occurs via the proliferation of tenocytes from the epitenon and endotenon producing mature collagen fibers, which results in less scar formation and fewer adhesions, while providing better mechanical strength [28]. Tendons with viable tenocytes and stable approximation might heal through intrinsic mechanisms. However, the exact role of intrinsic healing through direct tendon contact in AT ruptures is not well documented. Nonetheless, end-to-end direct contact and strong repair without a gap are still considered the most reliable methods to prevent AT lengthening, subsequent weakness, and re-rupture [7, 22, 29]. This reliability is at least partly due to the strong internal brace effect of the sutures, which allows enough time for the regenerated fibrous scar to remodel until it reaches sufficient strength without significantly compromising normal activity. In the PR group, although some of the ruptured fibers could be pulled together, the internal brace effect of the sutures was weaker compared to the 2MTIR group. Therefore, longer protection and more cautious rehabilitation with gradual exercises will be necessary, implying a greater medical burden. The technique used in both groups ended with knots at the junction of the two ends, a factor noted as a risk for skin complications or tendon elongation [7, 18, 30]. This is particularly plausible in the PR group, where the Bunnell suture might not create enough friction to hold the tendon securely. However no evidence of tendon lengthening was observed in the 2MTIR group.

In our series, we were less concerned about re-rupture in the 2MTIR group due to the superior primary fixation strength. Patients in the 2MTIR group were placed in a cast for four weeks and then began weight-bearing walking with a heel-raised walking boot. Most achieved a neutral ankle position by 5–6 weeks postoperatively and commenced full weight-bearing walking thereafter. In contrast, in the PR group, the cast was removed six weeks postoperatively, and then the same steps were followed as in the 2MTIR group. The difference in rehabilitation time could also contribute to irreversible muscle wastage, affecting the final heel rising strength [6]. The final results of strength and tightness also showed statistical differences, with less tightness and more weakness in the PR group, indicating more muscle waste or tendon lengthening in the PR group.

Blood supply preservation is better in the PR group than in the 2MTIR group. Despite the rupture stumps in the 2MTIR group being exposed and heavily sutured, no significant side effects were observed at the final follow-up. We believe circulation could reestablish from the undamaged paratenon to the avascular fibers within weeks in the 2MTIR group, and that extrinsic healing from the intact paratenon would not be significantly impaired.

No sural nerve injuries occurred in the 2MTIR group, while 7 out of 33 cases (21.2%) in the PR group experienced such injuries, showing a statistical difference. This could be due to direct needle puncture through the nerve or peripheral tissue compression or entrapment of the nerve. Sural neuritis after percutaneous procedures has been reported more frequently than with open techniques [31–34]. New improvements in percutaneous techniques, such as intraoperative real-time ultrasonography [16], exposure of the sural nerve [35] and longitudinal stab incision combined with repair under local anesthesia [14], have shown good results in avoiding nerve complications.

We observed no wound infections in either group. In both groups, the skin incision scars were minimal, and no obvious tendon-skin adhesion was noted when the AT was moving, indicating excellent paratenon preservation.

Our study found no statistical difference in the RTS level, even though subjective assessments of strength recovery showed significant differences. This discrepancy may necessitate further research into factors such as rehabilitation protocols and psychological changes post-injury. Recent developments in early-accelerated functional rehabilitation protocols have significantly improved the success of conservative treatment [8, 9, 23, 36]. The concept focuses on placing the ankle in a protective position while initiating muscle training and weight bearing soon after injury to prevent muscle atrophy and promote healing and functional recovery. However, this approach requires high patient compliance and careful, frequent monitoring and assessment [6, 37]. In our study, we focused more on surgical techniques and less on rehabilitation. Considering the results of the 2MTIR technique and the success of conservative treatments under the new concept of accelerated rehabilitation, we believe that a more aggressive, earlier rehabilitation approach should be applied to surgical repair cases to expect better recovery outcomes.

Finally, compared to other techniques, the 2MTIR approach is straightforward and can be easily performed by junior surgeons, consistently yielding good results. Our review of the literature did not reveal any documentation of a technique exactly like ours.

## Conclusion

The 2MTIR technique provided a technically straightforward, minimally invasive procedure with well-preserved paratenon and direct end-to-end firm fixation in cases of ACMATR. It resulted in very low complications, easy rehabilitation, and full weight-bearing as early as 5–6 weeks postoperatively, yielding better functional outcomes compared to the PR technique in the 1–5 year follow-up.

### Limitations of the study

The follow-up rate and the small number of cases may both introduce bias into the results. Additionally, all comparison indices are subjective, which might affect the reliability of the findings. Further studies with larger sample sizes and objective measures are recommended to validate these results.

## Data Availability

Original data has been uploaded in Related files.

## Abbreviations

ACMATR: Acute closed midsubstance Achilles tendon rupture
AT: Achilles tendon
2MTIR: Two mini transverse-incision repair
PR: Percutaneous repair
RTS: Return to Sports
